# Molecular Mechanism of Silicone Network Unbinding: Visco-Elastic Modes and Fracture upon Tensile Testing

**DOI:** 10.3390/polym18212688

**Published:** 2026-10-29

**Authors:** Pascal Puhlmann, Dirk Zahn

**Affiliations:** Lehrstuhl für Theoretische Chemie, Computer Chemie Centrum, Friedrich-Alexander Universität Erlangen-Nürnberg, Nägelsbachstraße 25, 91052 Erlangen, Germany

**Keywords:** silicone, tensile testing, molecular dynamics, network analyses

## Abstract

We report on tensile testing of atomistic simulation models mimicking silicone resins. To this end, we extend previous studies on silicone hardening and network formation (*Polymers* **2025**, *17*, 2619) to molecular dynamics simulations of the atomic-scale processes that account for elastic behavior, visco-elastic and plastic deformation. This encompasses Si–O bond scission and reformation, thus giving rise to siloxane network reorganization upon yielding, which implies both slipping of molecular chains and chemical modifications in terms of changing siloxane cross-linking. Based on graph theory, we discriminate percolating siloxane strands and elucidate the interplay of large per-atom stress in these load-bearing parts of the polymer network and heterogeneity of atomic displacements to dissipate local stress. On this basis, findings from atomic-scale characterization are discussed in the context of bulk polymer mechanical characteristics such as maximum stress, plasticity and fracture energy (4.7 ± 0.9 J/m^2^).

## 1. Introduction

Silicones based on polydimethylsiloxane (PDMS) cover a broad spectrum of properties and application possibilities. While PDMS formed from dimethylsilandiol (DMS-D) gives rise to linear chains, trivalent connectivity may be implemented by monomethylsilantriol (MMS-T) to achieve cross-linking of the –Si–O–Si– strands. Upon controlling the composition of the precursors, silicone formulation may hence be tuned from very soft elastomers (Young’s modulus Y = 1–100 MPa [1,2,3]) to stiff resins (Y = 2–4 GPa [4,5,6]).

Silicones prepared as soft elastomers feature siloxane networks with long and flexible chain segments among sparsely distributed cross-linkers. This is reflected by a glass transition temperature (T_g_ ~ 150 K) far below room temperature. Operating these materials above T_g_ implies soft, visco-elastic behavior, useful for applications such as rubber or reversible adhesives [7]. The mechanical behavior of such elastomers is comparably well understood, not least to established works like the Lake–Thomas theory of chain scission and network elasticity [8,9].

On the other hand, silicone resins are densely cross-linked and thus feature networks of comparably short linear chain segments. This implies a much higher glass transition temperature and offers resin formulations with T_g_ = 310–592 K [3,10,11,12,13,14] (T_g_ = 358 ± 5 K for the simulation model depicted in ref. [4]). For application as structural building parts and durable adhesives or sealants, the mechanical properties and fracture characteristics of silicone resins operated below T_g_ are of key importance [2,15,16,17]. Tuning the crosslinking density of silicone networks leads to a broad range of resins, whose tailor-made properties are reflected by an appealing range of technical applications. For example, silicone adhesives qualify for high-temperature conditions [1,2,18,19]; soft-robotics use silicone resins to balance flexibility and toughness [20,21,22,23]; and encapsulations in automotive, cryogenics and electric applications benefit from structural sealing to endure permanent and repeated loading [20,24,25].

The critical role of the network structure is furthermore demonstrated by the strong variation in mechanical failure and fracture characteristics in composites, cellular and foamed silicone resins [3,9]. While these materials feature meso-macroscale heterogeneity that offers in-depth characterization from microscopy, bulk silicone resins exhibit nm-scale heterogeneity which is experimentally more difficult to assess. Indeed, the fundamental processes of chain scission and reorganization occur at length and time scales elusive to conventional mechanical tests [26,27,28]. This motivates the elaboration of molecular simulation models to explore polymer fracture [29,30,31,32]. Going beyond idealized network models that pioneered this field, we recently developed specific setups that reflect the chemistry of organosilanol precursors and their reaction to form silicone oil, rubbers, and resins [4,33]. On this basis, an in-depth understanding of the atomic dynamics of silicone bulk materials may be achieved from 5 nm scale simulation cells.

Supported by graph theory, we demonstrated the nm-scale heterogeneity of siloxane connectivity and the percolation of strands through the (3D-periodic) simulation cell [4]. Using parallel simulation cells, a variety of percolating strand sizes, spatial orientations, overall number densities, and degree of lateral crosslinking density are achieved. In what follows, we use this platform for tensile testing molecular dynamics runs to elucidate the role of load-bearing (percolating) siloxane strands and coexisting network segments of lateral connectivity or even non-percolating character. To this end, we aim to bridge macroscopic characterization in terms of stress–strain profiles and microscopic understanding of bond scission, reformation, and molecular reorganization processes upon visco-elastic/plastic deformation and rupture.

## 2. Methods

Our simulations were performed on the basis of 3 independent models of bulk silicone. These starting points were depicted from our recent simulation study of dimethylsilandiol (DMS-D) and monomethylsilantriol (MMS-T) polymerization featuring silicone network formation from 90/10% DMS-D/MMS-T precursors [4]. Each system comprises 500 silanol moieties, forming percolating networks within 4 nm sized, 3D periodic simulation boxes. As a consequence of the heterogeneous network arrangement observed at the nanometer scale, the number of percolating polymer strands varies considerably, namely from 6 to 14, which calls for sampling averages from parallel setups to improve the statistical significance of our results. Apart from exploring 3 independent simulation cells, we performed the tensile testing molecular dynamics simulations in all 3 directions separately, thus providing a total of 9 runs.

Molecular dynamics simulations were performed with the LAMMPS package [34]. The integration time step was chosen as 1 fs. Periodic boundary conditions are applied in all directions. While the minimum image convention is used when re-scaling the simulation cell, all structural analyses are based on unwrapped coordinates. Relaxation of the simulation cell normal to the direction of tensile testing is allowed by means of 2D implementation of the Nosé–Hoover algorithm to impose 1 atm pressure and 300 K with barostat/thermostat coupling times of 1 ps and 0.1 ps, respectively [35]. To this end, tensile testing along the x-direction implies:

(1) $$L_{x}\left(t\right)=L_{x}\left(t=0\right)+v_{pull}\cdot t$$

whereas the box dimensions *L*_y_, *L*_z_ in the perpendicular directions are obtained from constant-pressure molecular dynamics simulations. For tensile testing molecular dynamics runs, high (indeed very high) strain rates have to be considered in the context of the nm-scale size of the simulation cells. To this end, implementing a strain rate of 10^8^ s^−1^ (as used in the production runs) refers to a pulling speed of *v_pull_* = 0.4 m·s^−1^ (Equation (1)). At each time step of our tensile testing molecular dynamics runs, increments in strain are applied by scaling the entire simulation cell. While this refers to ideal elastic deformation, atomic displacements related to plastic deformation and fracture are assessed from molecular dynamics simulations, i.e., by numerical integration of Newton’s equations of motion based on the forces acting on the individual atoms.

For describing the atomic interactions, including Si–O bond cleavage and possible re-formation, the underlying ‘reactive’ force-field was fully adopted from ref. [33]. A cutoff distance of 12 Å was employed for evaluating the real-space part of all pairwise interactions, whereas long-range electrostatic interactions were treated using the particle mesh Ewald summation method [36]. Python 3.12, NetworkX 3.3, and VMD 2.0 were used for data analysis and network analysis [37,38].

For the time-resolved characterization of the polymer network, we used graph-theoretical matrix representations to evaluate the –Si–O–Si– backbone. To identify Si–O bonds, we found a distance delimiter of 1.8 Å to be appropriate. The same delimiter is used for monitoring siloxane bond dissociation. This allows up to 0.2 Å elongation from the equilibrium distance of 1.6 Å, as experienced during thermal fluctuations and deformation within the elastic regime [4]. In turn, the displacements of atoms *i* were monitored by means of the squared deviation χ^2^ obtained from contrasting the actual atomic positions $\overset{⃑}{r_{i}}\left(t\right)$ to those expected from ideal elastic deformation. This provides a measure of the non-affinity of the atomic displacements:

(2) $${\chi_{i}\left(t\right)}^{2}={\left|\;\overset{⃑}{r_{i}}\left(t\right)-\hat{\epsilon }\left(t\right)\cdot \overset{⃑}{r_{i}}\left(t-∆t\right)\;\right|}^{2}$$

(3) $$\hat{\epsilon }\left(t\right)=\left(\begin{array}{ccc} \frac{L_{x}\left(t\right)}{L_{x}\left(t-∆t\right)} & 0 & 0\\ 0 & \frac{L_{y}\left(t\right)}{L_{y}\left(t-∆t\right)} & 0\\ 0 & 0 & \frac{L_{z}\left(t\right)}{L_{z}\left(t-∆t\right)} \end{array}\right)$$

where *L*_x_, *L*_y_, *L*_z_ refer to the dimensions of the simulation cell as functions of time *t*. To best illustrate local deviations from elastic deformation (linear scaling by $\hat{\epsilon }$), we found Δ*t* = 10 ps as a reasonable compromise for diminishing the effect of thermal fluctuations whilst still providing fine (10 ps) time resolution of the non-affinity measure.

While the non-affinity ${\chi_{i}}^{2}$ is used to contrast local differences observed for the displacement of individual atoms *i*, inspection of the dimensions of the simulation cell offers a more global assessment of elastic deformation characteristics versus unbinding, cavitation and crack propagation. To this end, we suggest overall volume conservation as indicative of the elastic regime, whilst volume increase proportional to the tensile deformation is related to crack nucleation and fracture. Thus, the limiting cases of elastic deformation and fracture are attributed to

(4) $$\left.\begin{array}{c} L_{x}\left(t\right)=L_{x}\left(t=0\right)+v_{pull}\cdot t\\ L_{x}\left(t\right)\cdot L_{y}\left(t\right)\cdot L_{z}\left(t\right)=constant \end{array}\right\}\to elastic\;deformation\;\left.\begin{array}{c} L_{x}\left(t\right)=L_{x}\left(t=0\right)+v_{pull}\cdot t\\ L_{y}\left(t\right)=const.\;L_{z}\left(t\right)=const. \end{array}\right\}\to crack\;propagation$$

To monitor the energetics of our model systems during tensile testing, we collected profiles of the potential energy based on the individual atomic interactions to describe the ‘inner energy’ of the polymer network. This is contrasted with the mechanical work *W_mech_* that arises from integration of the overall tensile stress *σ* upon model elongation by Δ*x*:

(5) $$W_{mech}=-\int_{0}^{\infty }\sigma \left(∆x\right)\cdot L_{y}\left(∆x\right)\cdot L_{z}\left(∆x\right)\;d\left(∆x\right)$$

## 3. Results

Before performing the tensile-testing molecular dynamics runs, we first elaborate the atom-scale characterization of our silicone resin models. Upon > 98% curing of 90/10% DMS-D/MMS-T precursors, the silicone network established in ref. [4] features 1–37 sized –Si(CH_3_)_2_–O– chains stemming from DMS-D, whereas the MMS-T crosslinkers provide 3D connectivity of the Si-O bonded network. In our 4 nm sized, 3D periodic simulation cell, we therefore find a series of percolating –Si–O– strands (featuring an average of 46 ± 18 siloxane moieties) that invoke covalent bonding throughout the boundaries of the simulation model. While the average number of percolating strands in a given direction was found to be 3.8 ± 1.4, it is intuitive to focus on the shortest percolating strand for exploring the local stress distribution during tensile deformation of the silicone network. To illustrate this, Figure 1 (left) highlights the shortest percolating strand along the horizontal direction.

The snapshot depicted in Figure 1 was actually taken before application of tensile testing along the x-direction. To this end, the overall stress of the system is zero. However, for individual siloxane units we find the local stress to differ quite considerably from the overall average. Using the silicon atoms for representation of the mono-/dimethylsiloxane moieties, the corresponding per-atom stresses were summed up and mapped on Si by means of a color code (Figure 1, center). In turn, the underlying occurrence profile of local stress experienced per siloxane moiety is provided in Figure 1, right. For comparison, we also computed the distribution of local stress values for the percolating strand featuring the shortest covalent connection along the horizontal direction (Figure 1, left).

Indeed, for all percolations we observe a trend towards negative per-atom stress—indicating tensile stress on these strands—whilst the remaining system experiences compressive stress such that the overall stress of the model system cancels out. Inspection of all percolating strands showed an average of −0.16 MPa per siloxane moiety, whereas siloxanes in the remaining system experience about +0.07 MPa. To this end, about 30% of the siloxane groups are involved in percolating strands and experience tensile stress summing up to −24.3 MPa. In turn, 70% of the siloxanes are located in lateral loops and branches, which experience compressive stress such that the overall resin is at equilibrium. This implies a remarkable heterogeneity at the nm scale—which, in what follows, is related to plastic deformation and crack nucleation upon tensile testing of the silicone resin models.

Tensile testing molecular dynamics runs were performed for three independent simulation cells, all adopted from ref. [4]. For each system, we explored deformation along the *x*, *y* and *z* directions in separate runs. When exploring structural and elastic properties in ref. [4], we found that the number of percolating strands, Young’s moduli, etc., observed for *x*, *y* and *z* directions fully reflect the variety observed when comparing different model systems. On this basis, we argue that the overall set of 3 × 3 simulation runs mimics nine independent tensile testing setups. We tested a small series of strain rates of 10^10^, 10^9^ and 10^8^ s^−1^ to illustrate the role of pulling at a speed of 40, 4 and 0.4 m/s, respectively. As an example of tensile testing along the horizontal direction of the model illustrated in Figure 1, the stress–strain profiles are shown as a function of strain rates in Figure 2 (left). For model elongation within the range Δ*x* = 0–5 nm, we find reasonable convergence of the stress–strain profiles upon reducing the strain rate to 10^8^ s^−1^. This includes the first event of bond cleavage in the silicone resin model at Δ*x* = 1.9 nm and subsequent relaxation of the polymer network (as indicated by the sharp decline of system stress, Figure 2, left). For the cascade of further bond dissociation events, we indeed observe an increasing difference in the stress–strain diagrams as functions of pulling speed. However, this appears well within the variety of stress–strain diagrams when comparing different resin models (and different pulling directions). Based on our slowest tensile testing setup, Figure 2 (right) shows the stress–strain diagrams stemming from models that showed the first bond cleavage event at a particularly early stage (Δ*x_first_* = 1.9 nm)—as compared to an average scenario (Δ*x_first_* = 7.5 nm) and particularly late –Si–O– bond dissociation, respectively. Considering this variety, we argue that the deviation of the stress–strain diagrams obtained for *v*_pull_ = 4 and 0.4 m/s appears acceptable. To this end, selection of a strain rate of 10^9^ s^−1^ could be justified. However, to be on the safe side, we decided on a strain rate of 10^8^ s^−1^—and likewise *v*_pull_ = 0.4 m/s—for our production runs.

To elucidate the mechanisms of deformation and fracture, we combine our analysis of the stress–strain diagrams with atomic-scale characterization of the underlying simulation models. This is illustrated for the silicone resin depicted in Figure 1, namely for tensile testing along the horizontal direction. To this end, it is useful to plot stress *σ*(Δ*x*) and the non-affinity measure $\chi^{2}\left(∆x\right)=\frac{1}{N}\sum_{i=1}^{N}{\chi_{i}}^{2}\left(∆x\right)$ (see also Equations (2) and (3)) as functions of model elongation Δ*x* (Figure 3, top). To avoid bias from human intuition, thermal motion has not been separated from structural rearrangements. However, the extent of thermal noise may be concluded from the non-affinity measure near Δ*x* = 0. On this basis, we can attribute < 0.01 nm^2^ to thermal fluctuations, whilst abrupt stress relaxation leads to (momentous) increases in atomic displacements that are clearly non-affine to elastic deformation (i.e., homogeneous scaling of the atomic coordinates). Interestingly, the first bond cleavage event (A, Δ*x* = 2.0 nm) implies only a moderate increase in the overall non-affinity averaged over the entire simulation cell. While A is obviously beyond the elastic regime, we argue that the early stages of visco-elastic/plastic deformation largely retain the overall polymer network structure—and sacrifice only individual siloxane strands to release some of the momentous stress. This picture is reflected by the roughly constant profile of the box volume (Figure 3, bottom) upon tensile testing up to a second critical event (B, Δ*x* = 8.1 nm). Interestingly, we find several bond-rupture events before the silicone resin model shows non-constant volume as a function of elongation Δ*x*. At this stage (Δ*x* > 8.1 nm), the box dimensions normal to the pulling direction are roughly constant. Finally, (C, Δ*x* = 18.6 nm) denotes the rupture of the last percolating siloxane strand and hence the fragmentation of the resin into two pieces.

On this basis, we suggest three critical events as indicative of the deformation and fracture mechanisms. After leaving the elastic regime, our silicone resin model may sustain a limited number of –Si–O– bond dissociation events without a drastic signature of non-affine atomic displacement (as compared to elastic deformation), keeping the overall volume of the simulation cell constant. This suggests a large degree of visco-elastic deformation between snapshots A and B. In turn, constant cross-sectional area normal to the pulling direction (Equation (4)) and non-constant volume are identified for the increased non-affinity between snapshots B and C. At this stage, our model obviously experiences plastic deformation—along with crack nucleation and propagation.

To discriminate the different behavior of the underlying milestones A and B, we probed sample relaxation upon removal of external constraints. For this, we depicted snapshots taken 0.25 nm before and after the actual bond dissociation events. Accordingly, we inspect the silicone resin deformation at a constant pulling speed up to Δ*x*(A) − 0.25 nm = 1.75 nm, that is, before the first –Si–O– bond dissociation event, and explore structural relaxation from constant-pressure (1 atm), constant-temperature (300 K) MD simulation. Within ns-scale relaxation, we find the model to relax by −1.1 nm, which accounts for 63% of the strain applied before releasing the sample (Figure 4, left). In turn, when implementing analogous relaxation starting from tensile deformation up to Δ*x*(A) + 0.25 nm = 2.25 nm, we find the sample dimension along the horizontal direction to be reduced by only −0.3 nm—or 13% of the initially applied strain. On this basis, we attribute the milestone A to a shift from visco-elastic to plastic deformation. To this end, –Si–O– bond scission clearly relates to the mechanism of plastic deformation, whereas shearing and slipping of polymer strands is suggested to account (visco-)elastic deformation of the silicone network.

While the two percentages clearly differ, we point out that we do not observe a clear 100%/0% relation. Accordingly, we expect that modes of visco-elastic deformation also apply to stages of tensile testing beyond milestone A. To elucidate this, we looked into sample relaxation before/after milestone B. Free propagation started from Δ*x*(B) − 0.25 nm = 7.85 nm and Δ*x*(B) + 0.25 nm = 8.35 nm showed 31% (−2.4 nm) and 24% (−2.0 nm) recovery of the elongation Δ*x*, respectively. To this end, the difference in percentages for recovery of sample recovery diminishes from 50% near A to 7% near B, respectively. This trend carries on through the plastic deformation regime between B and C. Indeed, we find even smaller differences in the percentages of recovering sample elongation, e.g., 46.7% and 44.8% at Δ*x* = 10.75 and Δ*x* = 11.25 nm, respectively, as compared to 49.2% and 49.0% at Δ*x* = 12.75 and Δ*x* = 13.25 nm, respectively.

The distribution of local stress and non-affinity of the siloxane moieties upon tensile testing is illustrated in Figure 5. For milestone A, which is the first rupture event of a percolating strand, we clearly identify large per-atom stress before –Si–O– bond scission and an abrupt reduction in local stress thereafter. This is accompanied by a sharp increase in the non-affinity measure applied to the siloxane moieties of the breaking polymer strand. For milestone B, such heterogeneous system relaxation is also observed, albeit to a less pronounced extent. In line with the relaxation analyses discussed before, we argue that the differences before/after rupture of individual polymer strands are largest for A because of the deformation mechanism evolving from largely visco-elastic behavior to the onset of plastic deformation modes. Likewise, we attribute bond scission observed at milestone B to a regime of predominantly plastic deformation modes.

Finally, milestone C is depicted as the rupture of the last polymer strand (Figure 5, bottom). Apart from the –Si–O– bond dissociation events leading to the separation of all percolating siloxane strands in the pulling direction, the overall polymer network experiences drastic rearrangements in terms of slipping loops and even the formation of new –Si–O– bonds. Indeed, the majority of broken siloxane bonds do not lead to dangling Si and O atoms but, upon structural relaxation of the silicone resin, give rise to the formation of new network nodes. This phenomenon is dramatically reflected by the energy profiles assessed for the tensile testing runs.

In Figure 6, we contrast profiles of the mechanical work applied during sample deformation (based on the integral of the stress–strain diagram) and the potential energy of the simulation system with the cumulative counts of –Si–O– bond dissociation and formation events. For sample elongation, say less than 0.04 nm (1% strain), we find the internal energy of the simulation system identical to the externally applied mechanical work within 8% margins. Such coherence of the two energy profiles indicates elastic behavior. Upon the onset of visco-elastic modes, the slipping of polymer loops accounts for local relaxation that may involve energy gradients normal to the pulling direction. To this end, the integral of the restoring force and internal energy of the simulation system begins to deviate. Likewise, the reorganization of siloxane bonds provokes strong deviation between the two energy profiles. While scission of –Si–O– bonds upon pulling is reflected in both the externally applied work and the system energy, the formation of new siloxane bonds often implies lateral connections. Such newly formed lateral connections clearly reduce potential energy (as compared to dangling –Si and –O moieties), as indicated by the sharp decline in the potential energy profile. The underlying processes and likewise the related energy gradients are, however, not perfectly parallel to the pulling direction. Accordingly, the restoring forces to tensile deformation are somewhat reduced, but never become negative such that the externally applied mechanical work (Equation (5)) does not decline.

Based on the three different siloxane resin models and three independent pulling directions, we collected averages of the Young’s modulus *Y* = 3.2 ± 0.4 GPa and the fracture energy *W_mech_*(Δ*x*→∞) = 4.7 ± 0.9 J/m^2^, respectively. For the different tensile testing runs performed, both of these properties vary by up to 50% of the mean values. This reflects the small size of the simulation model and likewise the importance of nm-scale heterogeneity in the polymer network. To this end, averages taken over many small models are needed to mimic the mechanical properties of meso- to macroscale-sized samples. However, it is intriguing to also use our collection of data sets from nm-sized simulation cells for inspecting the correlation of the different properties assessable to our molecular dynamics runs.

Along those lines, we first investigated possible correlation of fracture energy and Young’s modulus against structural data, such as the number density of percolating siloxane strands in the resin. However, upon attempting linear regression, we find rather poor correlation (showing regression coefficients of R^2^ < 0.4). Considering the complexity of our siloxane resin network model, it appears quite intuitive that simple structure-property relations are not within reach [4]. Likewise, the Young’s modulus and the work of fracture showed only weak correlation (Figure 7). In view of the importance of heterogeneous modes of displacement of the siloxane polymer strands during tensile deformation, we furthermore investigated the relation of critical events regarding –Si–O– bond scission to Young’s modulus and fracture energy. For this, we focused on the role of the sample elongation Δ*x_first_* and Δ*x_last_* for which the tensile testing runs showed the first and the last –Si–O– bond rupture events, respectively. While there is no correlation of either Δ*x_first_* or Δ*x_last_* to the Young’s modulus, for *W_mech_* we indeed observe a linear relation to the final rupture event at Δ*x_last_* (Figure 7).

On this basis, we suggest that both the Young’s modulus and Δ*x_last_* are relevant descriptors of the fracture energy of our silicone resin models. Interestingly, *Y_xx_* reflects the stiffness of the material and may hence be attributed to aspects of brittle behavior. In turn, the elongation Δ*x_last_* at the final –Si–O– rupture event is clearly connected to visco-elastic and plastic modes of deformation and fracture. To this end, a combination of different mechanistic aspects is required to rationalize the fracture mechanics of the silicone network.

## 4. Conclusions

The complexity of silicone resins poses considerable challenges to all-atom molecular dynamics simulations. On the one hand, it is far from trivial to set up realistic simulation models that mimic the underlying silicone network at a minimum of human prejudice. While this issue was addressed in a previous study [4], in the present work we demonstrated the implementation of tensile testing runs and elucidated the interplay of local events and overall characteristics of sample deformation and fracture. On this basis, we suggest the combination of atom-scale measures and overall potential energy profiles for the rationalization of stress–strain or stress–elongation diagrams. Apart from per-atom stress, we unravel local heterogeneity by a non-affinity measure that quantifies the discrepancy of atomic displacements with respect to ideal elastic deformation. This is complemented by inspection of –Si–O– bond dissociation events and monitoring of new bonding of the otherwise dangling –Si and O– moieties.

The rupture of covalent bonds clearly marks the most obvious aspect of plastic deformation and fracture. The underlying relaxation of local stress reduces the restoring forces opposing sample deformation and thus affects the curvature of the profiles of the work of fracture as a function of sample elongation. In terms of the internal energy of our simulation systems, however, we find a more dramatic effect, namely a sharp decline in potential energy caused by the formation of new bonds. Indeed, scission of polymer strands leads to a considerable degree of re-connecting of the silicone network. To this end, visco-elastic and plastic deformation modes are attributed to the rearrangement of the polymer network not only by slipping of polymer strands, but also by changing overall connectivity of the siloxanes.

These mechanisms were observed throughout a small series of independent molecular dynamics runs, and we argue that both the mechanistic concepts and the elaborated protocols for analyzing them appear of wider relevance to silicone resins. However, we caution that our 4 nm sized simulation cells limit the quantitative prediction of both structural and mechanical data. This is reflected by the variety in the number of percolating polymer strands monitored for the different tensile testing setups and likewise the range of model elongations observed upon first (∆x(A) = 2.0–13.4 nm) and final (∆x(C) = 9.9–38.4 nm) Si–O bond scission events, respectively.

For our silicone resin models based on 90/10% DMS-D/MMS-T precursors reacted to a curing degree of 98–99%, the work of fracture was found to be 4.7 ± 0.9 J/m^2^. While no direct experimental counterpart of our specific silicone resin system could be found in the literature, the ballpark of commercial resins reflects the tuning of fracture energy within a large range (the closest match in terms of fracture energy, e.g., 6.5 ± 0.2 J/m^2^, was observed for L182 silicone adhesives, which actually feature rather low crosslinking density [19]; however, upon large crosslinker content, even 10^3^ J/m^2^ scale fracture energy may be achieved [16,19,39,40,41,42,43]).

## Figures and Tables

**Figure 1:**
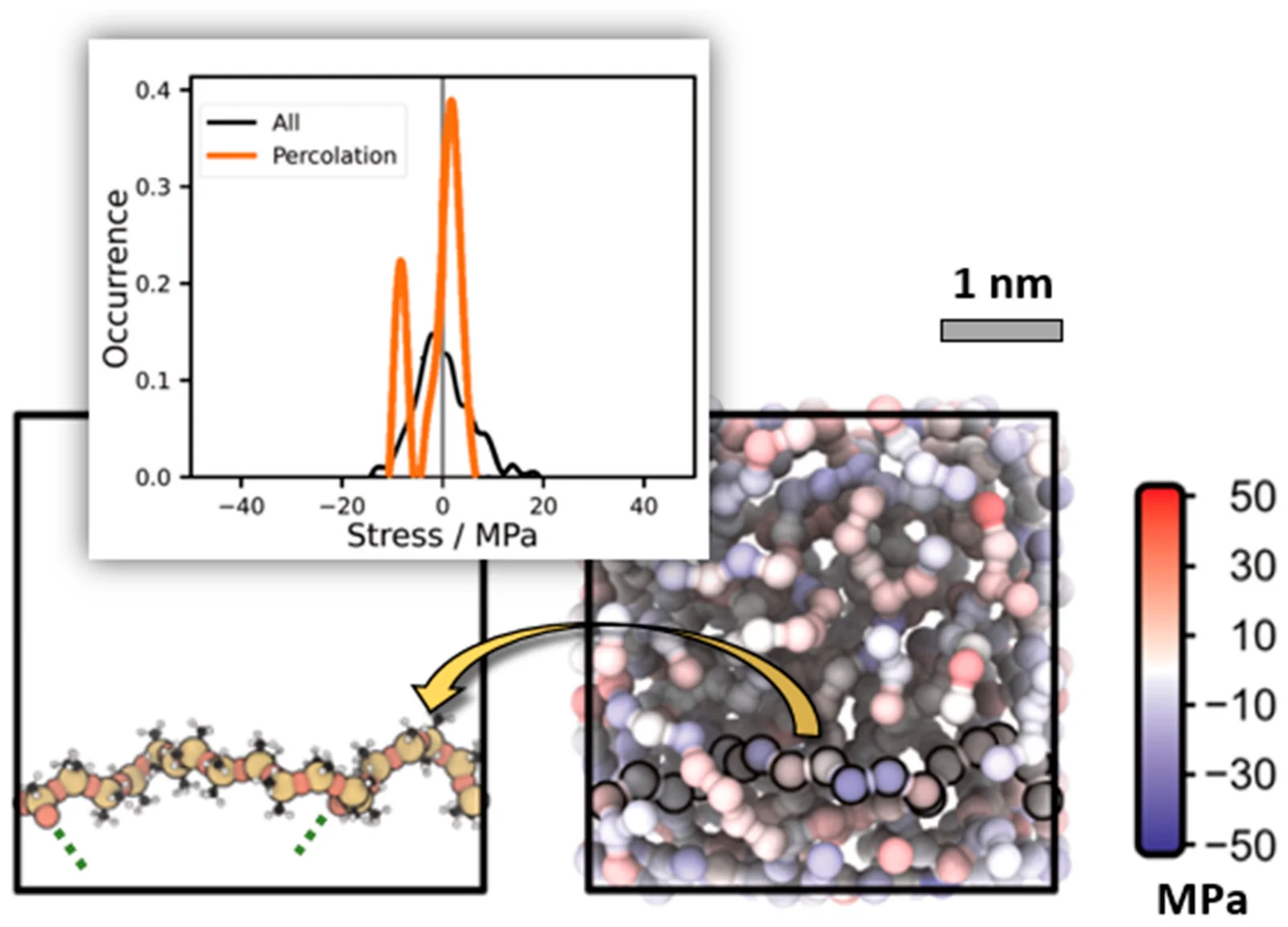
Illustration of local stress distribution ((**top**) occurrence profiles, (**lower right**) spatial distribution) in the 3D-periodic simulation cell mimicking the silicone resin (500 siloxane groups, 90% DMS-D and 10% MMS-T, curing degree of 99%). To diminish possible effects of thermal fluctuations, averages were sampled from a 10 ns MD run at 300 K and 1 atm. For clarity, at the lower right we show only the Si atoms using a color code to illustrate the local stress acting on the underlying siloxane moiety. The highlighted strand reflects the shortest percolation along the horizontal direction. At the lower left, this chain of –Si–O– bonds is shown separately (Si: brown, O: red), including also the –CH_3_ groups of the monomethyl- and dimethylsiloxane building blocks. Likewise, the oxygen atoms providing crosslinking of MMS-T are indicated (dotted lines).

**Figure 2:**
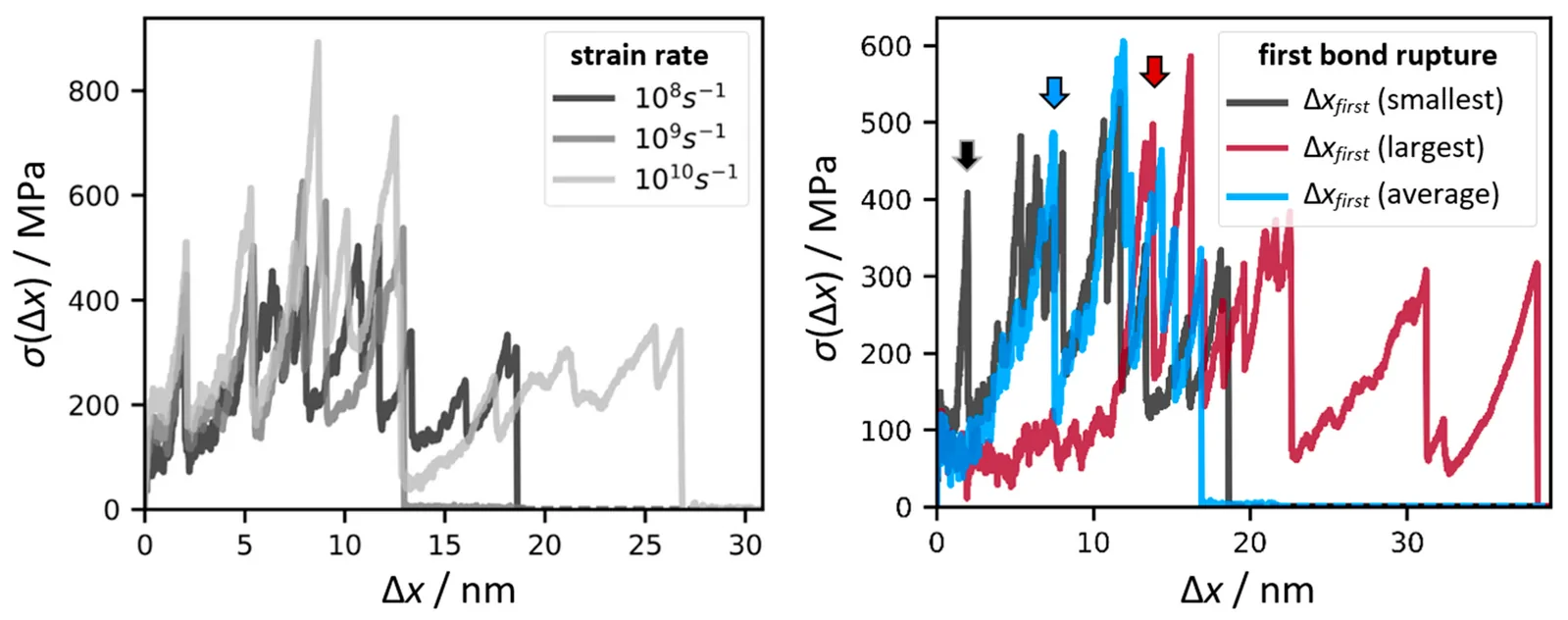
(**Left**) Stress–strain diagrams for tensile testing molecular dynamics at different strain rates. Using identical simulation cells as starting points we find good agreement for elastic deformation and the early stages of plastic deformation, as indicated by the first –Si–O– bond rupture event. (**Right**) Comparison of tensile testing runs performed at the slowest strain rate explored (10^8^ s^−1^). The depicted systems feature siloxane bond dissociation at different stages. This comprises the smallest and largest elongation at first bond rupture (Δ*x_first_* indicated by the arrows) we found from our series of 9 tensile testing runs—along with a representative curve illustrating the average picture.

**Figure 3:**
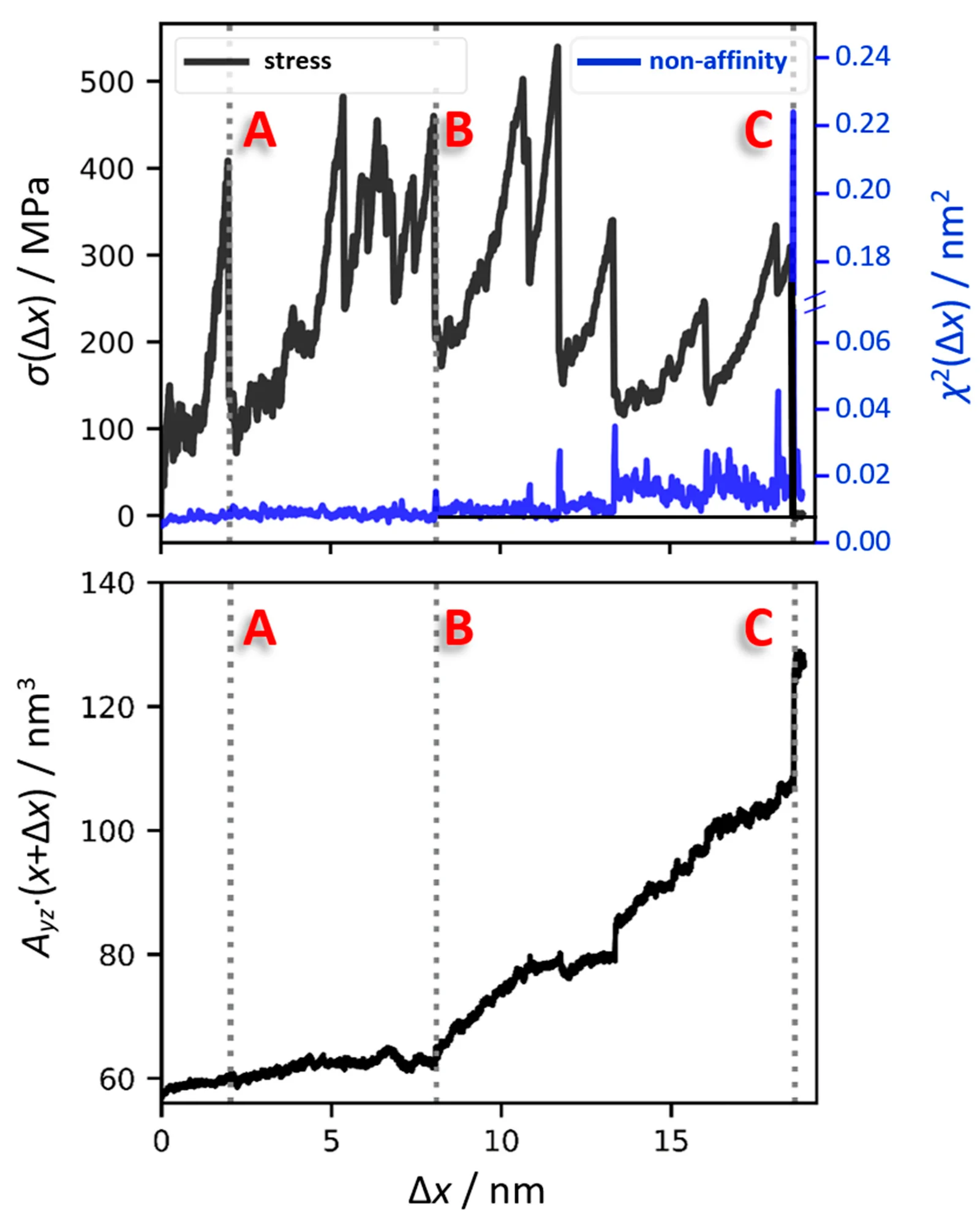
(**Top**) Stress–strain diagram and non-affinity measure for tensile testing molecular dynamics simulation of the silicone resin model illustrated in Figure 1. For the mechanistic discussion, we identify key intermediate structures: A—the first –Si–O– bond rupture; B—the event of bond scission giving rise to box deformation (**bottom**) normal to the pulling direction; C—the final fracture leading to two separate fragments. Each bond dissociation event is indicated by a sharp decrease in system stress, leading to polymer network relaxation. For such partially relaxed structures, the stress reduces to a roughly constant plateau within 100–200 MPa.

**Figure 4:**
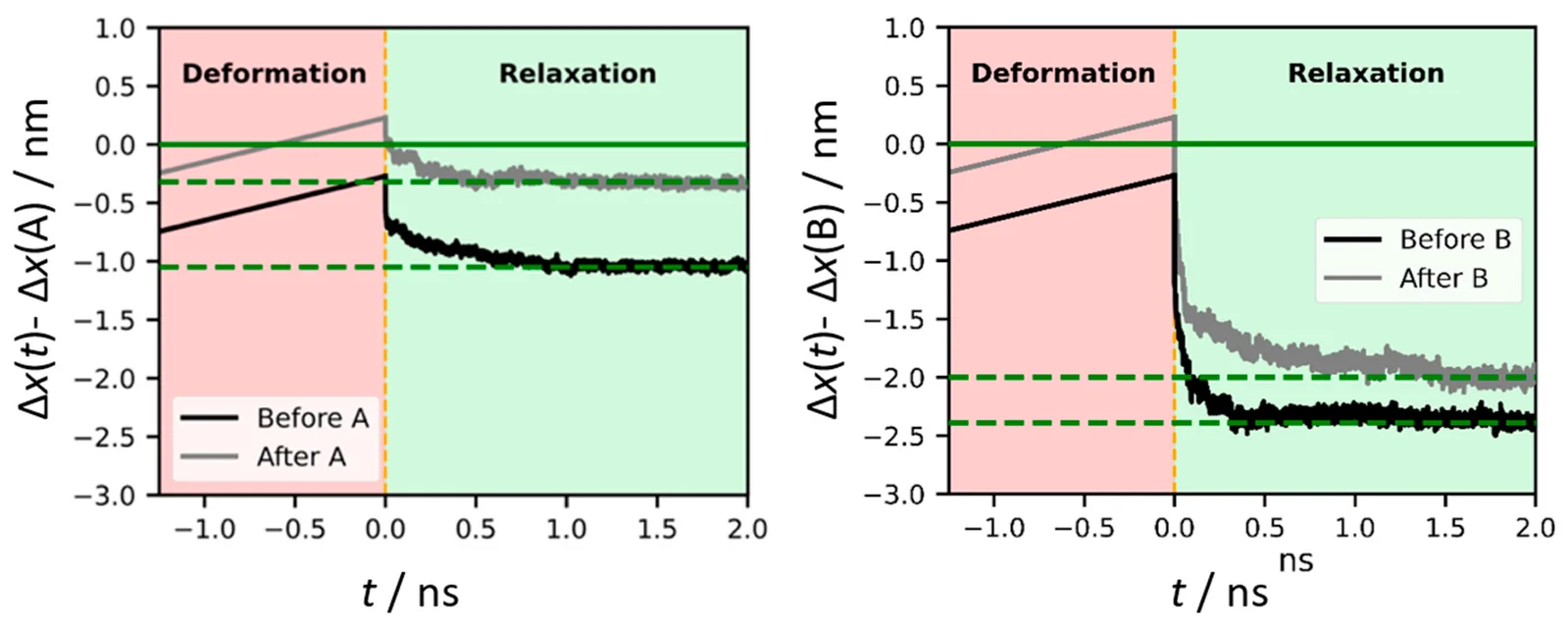
(**Left**) Silicone resin relaxation after tensile testing: molecular dynamics runs up to 0.25 nm before/after milestone A, i.e., the first –Si–O– bond dissociation event. Sample elongation *x*(t) as a function of time is shown for the deformation process (t < 0) and upon free relaxation starting at t = 0. Shape recovery of the sample before/after milestone A differs significantly. (**Right**) Analogous runs performed before/after milestone B. Shape recovery of the simulation box upon free relaxation initiated from snapshots before/after milestone B shows only marginal differences.

**Figure 5:**
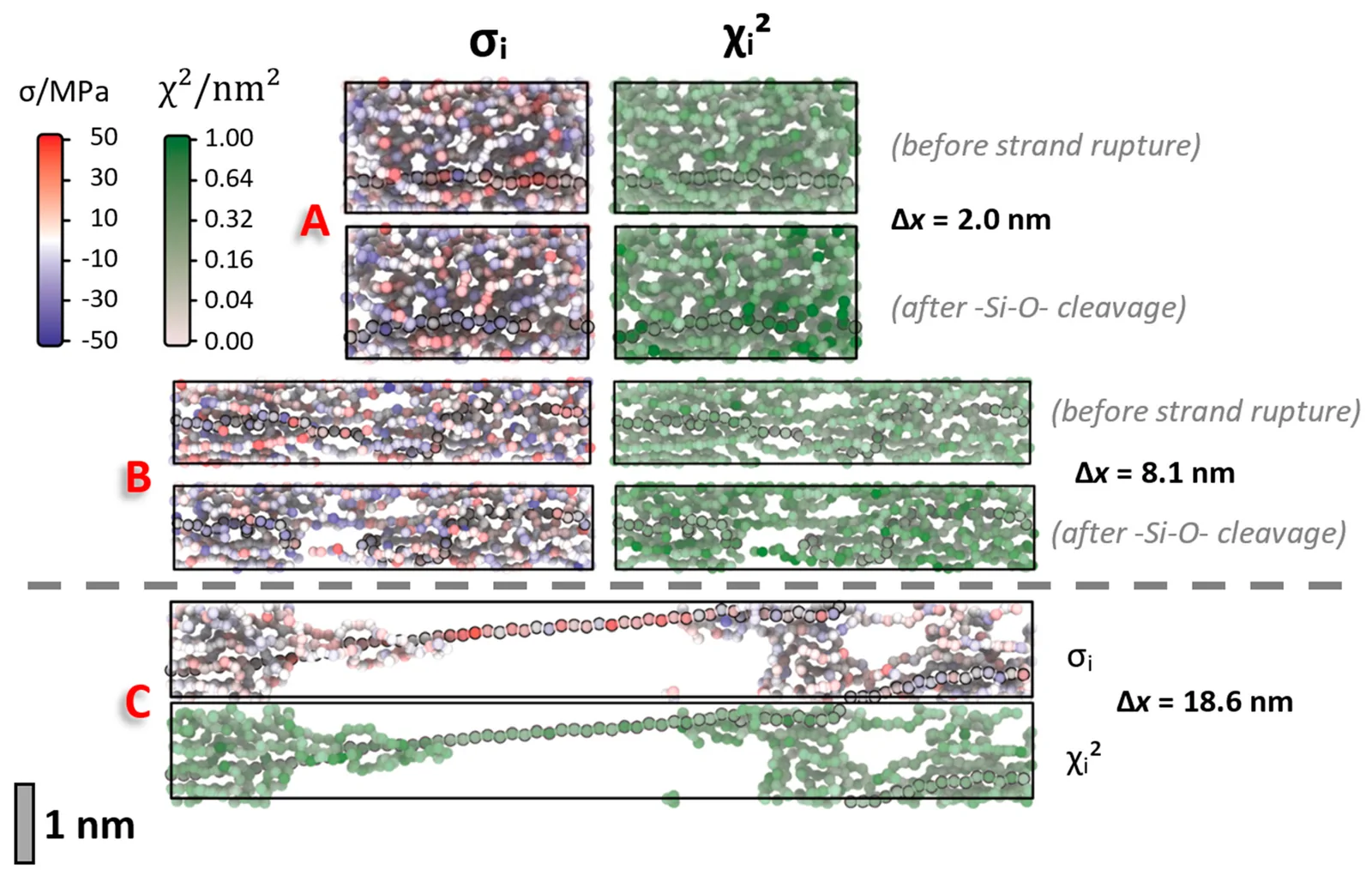
Illustration of local stress σ_i_ and non-affinity χ_i_^2^ per siloxane moiety *i* for the milestones A, B and C depicted for the tensile testing run highlighted in Figure 3. Even before the rupture of covalent bonds, polymer strands undergo slipping/sliding events as indicated by large non-affinity up to χ^2^ = 1 nm^2^. For the first –Si–O– bond dissociation event A and that of the interim milestone B, we show snapshots 10 ps (**upper** row) before and 10 ps after (**lower** row) bond dissociation. Milestone C marks the final bond cleavage, and color-coding of per-atom stress and non-affinity is thus only meaningful before rupture.

**Figure 6:**
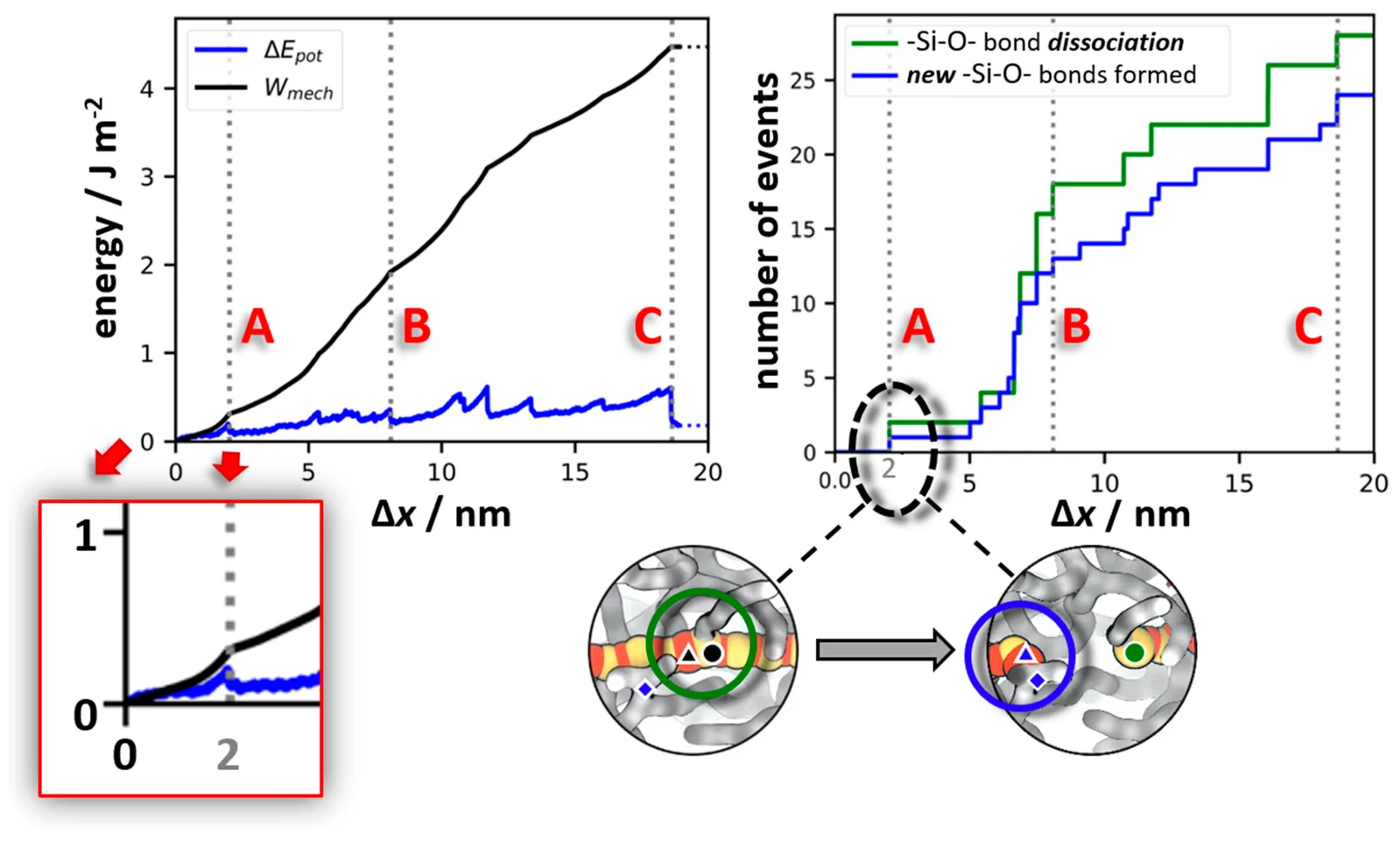
Profiles of key descriptors explored during tensile testing. (**Left**) (externally applied) mechanical work and the potential energy of the simulation system (stemming from internal interactions) as functions of box elongation Δ*x*. (**Right**) cumulative sum of –Si–O– bond scission and formation events. While the mechanical work experienced during tensile testing is attributed to bond elongation and rupture, the formation of new –Si–O– bonds is reflected only by a sharp reduction in the potential energy. For illustration, the bond scission occurring at milestone A is depicted by the snapshots shown at the lower right. Here, fragmentation of a percolating strand leads to a dangling Si atom (green dot), whereas the –O moiety experiences association with a lateral siloxane group.

**Figure 7:**
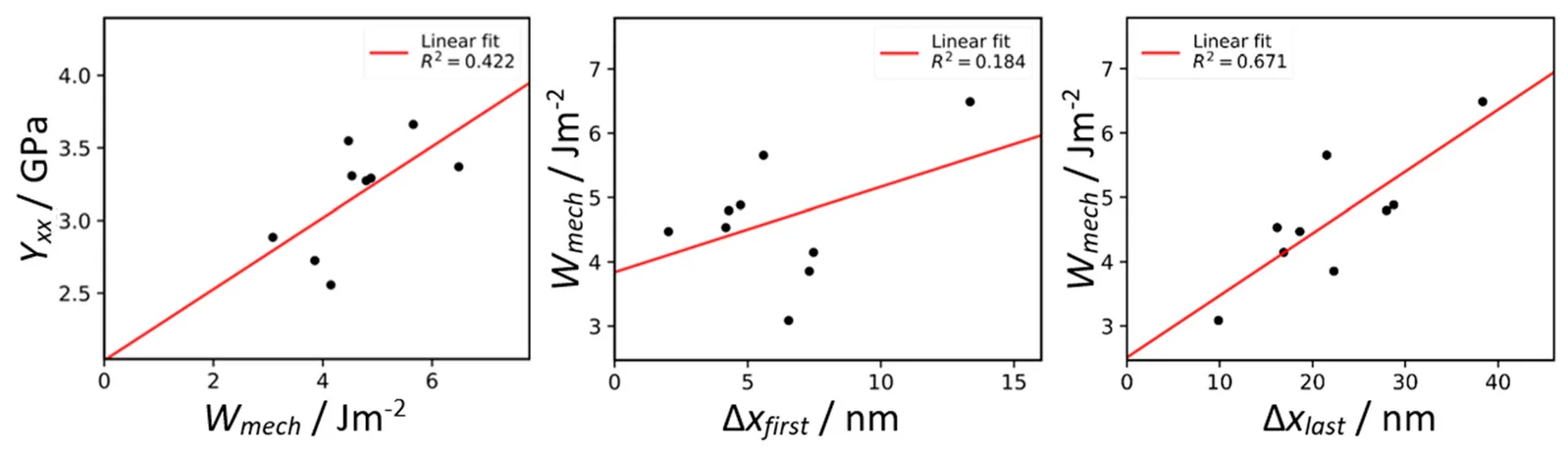
Correlation analyses of mechanical properties collected from our series of 9 independent tensile testing runs. (**Left**) work of fracture *W_mech_* as compared to the Young’s modulus *Y_xx_* assessed along the pulling direction. (**Center**) *W_mech_* shows no correlation to the sample elongation Δ*x_first_* at the instant of first –Si–O– bond scission. (**Right**) roughly linear correlation of *W_mech_* and the sample elongation Δ*x_last_* at the instant of the final –Si–O– bond scission event.

## Data Availability

Raw data available upon request to the authors.
